# Preparation and Photocatalytic Performance of Hollow Structure LiNb_3_O_8_ Photocatalysts

**DOI:** 10.1186/s11671-017-2291-6

**Published:** 2017-09-02

**Authors:** Haifa Zhai, Jingjing Qi, Xiang Zhang, Hongjing Li, Liping Yang, Chunjie Hu, Hairui Liu, Jien Yang

**Affiliations:** 1 Henan Key Laboratory of Photovoltaic Materials, College of Physics and Materials Science, Henan Normal University, Xinxiang, 453007 People’s Republic of China; 2 National Laboratory of Solid State Microstructures, Nanjing University, Nanjing, 210093 People’s Republic of China

**Keywords:** LiNb_3_O_8_, Photocatalyst, Hollow structure, Hydrothermal

## Abstract

Hollow structure LiNb_3_O_8_ photocatalysts were prepared by a hydrothermal method assisting sintering process. The particles’ aggregation to form hollow structures with obvious cavities can be attributed to the Li element volatilization during calcination process. All the LiNb_3_O_8_ powders show high photocatalytic efficiency of degradation of methylene blue (MB), especially for the sample calcined at 700 °C (LNO700), with only 3 h to completely decompose MB. The photo-degradation of MB follows the pseudo-first-order kinetics, and the obtained first-order rate is 0.97/h. The larger degradation rate of LNO700 can be attributed to its hollow structure which provides a larger specific surface area and more active sites to degrade the MB molecules. The cycling test of photo-degradation and adsorption of MB over LNO700 powder indicates that the hollow structure of the LiNb_3_O_8_ photocatalyst is stable and the LiNb_3_O_8_ photocatalyst is an efficient photocatalyst with good reusability, confirmed by the XRD and X-ray photoelectron spectroscopy tests before and after photo-degradation of MB.

## Background

Recent years, energy crisis and environmental pollution have become two urgent challenges, which seriously inhibit the economic development and human health. Photocatalysis is considered as the answer to the both problems, as it has the ability to produce hydrogen and decompose organic pollutants. Since Fujishima and Honda discovered the photocatalytic splitting of water using TiO_2_ as an electrode in 1972 [1], TiO_2_ has been widely investigated in the degradation of organic pollutions in water. Since then, various semiconductor materials are studied to search for the most convenient photocatalyst with high efficiency, low cost, environmental friendliness, and direct utilization of sunlight.

Niobates, mainly including three groups: alkali niobates, columbite niobates, and rare-earths orthoniobates, have been widely studied in many applications such as optical devices, solid electrolytic capacitors, dye-sensitized solar cells, and catalysis due to their interesting physical and chemical properties [2–4]. For the applications of clean energy and environmental remediation, some niobates, such as BiNbO_4_ [5, 6], LiNbO_3_ [7], (Na, K)NbO_3_ [8], and LiNb_3_O_8_ [9–15], have been investigated due to their unique distorted [NbO6] octahedral structures which provide active sites for photocatalysis. Among these materials, LiNb_3_O_8_ is considered as a novel lithium-ion battery (LIB) anode material with a large theoretical capacity of 389 mAh/g assuming two-electron transfers (Nb^5+^→Nb^3+^) [10, 11]. As a photocatalyst, LiNb_3_O_8_ shows efficient production of hydrogen and degradation of the organic pollutant of toluidine blue O (TBO) [12–14].

The conventional preparation method of niobates is solid-state reaction, while it always results in an inhomogeneous distribution of Li element in the preparation of Li-Nb-O compounds due to the easy volatilization of Li element at high annealing temperature. At most time, LiNb_3_O_8_ is easily formed and recognized as an impurity phase during the preparation of LiNbO_3_. Compared to solid-state reaction, the hydrothermal method is widely used to synthesize nanomaterials with a small particle size, which could provide a larger specific surface area and more active sites in the applications, especially for the photocatalytic process. Hollow structures, always accompanied with excellent performances, have attracted much attention and have been used in many fields, such as catalysis [16]. Great efforts have been made to improve the photocatalytic activity of semiconductors with various porous and hollow textures, as the hollow structure can not only lead to a higher specific area but also increase light-harvesting efficiency due to multi-scatting of light [17–23]. For a hollow structure LiNb_3_O_8_ photocatalyst, there is still no report before and the research of LiNb_3_O_8_ is still rare.

In this paper, hollow structure LiNb_3_O_8_ photocatalysts were prepared by the hydrothermal method assisting sintering process. The crystal structures, microstructures, and optical properties were studied systematically. The photocatalytic performance of hollow structure LiNb_3_O_8_ photocatalysts was evaluated by the degradation of methylene blue (MB) under UV light irradiation.

## Methods

### Photocatalyst Preparation

Hollow structure LiNb_3_O_8_ photocatalysts were prepared by the hydrothermal method assisting sintering process using lithium hydroxide monohydrate (LiOH·H_2_O, Aladdin, ACS, ≥ 98.0%) and niobium pentaoxide (Nb_2_O_5_, Aladdin, AR, 99.9%) as raw materials without further purification. Firstly, 3.5 mmol of Nb_2_O_5_ was dispersed into 35 mL deionized water with a certain amount of LiOH·H_2_O (the mole ratio of Li:Nb = 8:1) added under magnetic stirring for 1 h. Then, the suspension solution was put into a 50-mL Teflon-lined hydrothermal synthesis autoclave reactor and maintained at 260 °C for 24 h. After cooling down to room temperature naturally, the obtained white powders were centrifuged, washed with deionized water, and dried. Finally, the powders were calcined at various temperatures from 600 to 1000 °C for 2 h with a ramp rate of 5 °C/min.

### Characterization

The crystal structures of LiNb_3_O_8_ powders were analyzed using X-ray powder diffraction (XRD, Bruker D8 Discover) with Cu Kα radiation. The morphologies of the powders were characterized by field emission scanning electron microscopy (SEM, JSM-6700F) and the chemical composition was measured by energy dispersive X-ray spectroscopy (EDS) performed in SEM. The UV-vis diffuse reflectance spectra (DRS) of the powders were recorded by a UV-vis-NIR spectrophotometer (UV-3600, Shimadzu). The photoluminescence (PL) spectra were detected using a Jasco FP-6500 fluorescence spectrophotometer. The specific surface area was measured on a surface area apparatus (Micromeritics ASAP 2460) at 77 K by N_2_ adsorption/desorption method (BET method). X-ray photoelectron spectroscopy (XPS) analysis was performed on a Thermo-Fisher Escalab 250Xi instrument.

### Catalytic Tests

To evaluate the photocatalytic performance of the hollow structure LiNb_3_O_8_ photocatalysts, the degradation of MB aqueous solution (10 mg/L) was carried out under irradiation of a 500-W Hg lamp at a natural pH value. Fifty milligrams of powders were dispersed into 50 mL of MB aqueous solution. Before the irradiation, the suspension was kept in the dark for 1 h under stirring to achieve adsorption equilibrium. Then, the suspension was irradiated by the Hg lamp, and the residual concentration of MB was analyzed using UV-3600 at 665 nm with an interval of 1 h. In addition, the total organic carbon (TOC) of the mixture was determined via using a high-TOC Elementar Analyzer system to investigate whether the dye is completely degraded.

To detect the active species during photocatalytic reactivity, electrons (e^−^), holes (h^+^), hydroxyl radicals (·OH), and the superoxide radical (O_2_^·−^) were investigated by adding 5 mM AgNO_3_ (a quencher of e^−^), EDTA-2Na (a quencher of h^+^), tert-butyl alcohol (*t*-BuOH, a quencher of ·OH), and benzoquinone (BQ, a quencher of O_2_^·^), respectively. The method was similar to the former photocatalytic activity test.

## Results and Discussion

**Fig. 1:**
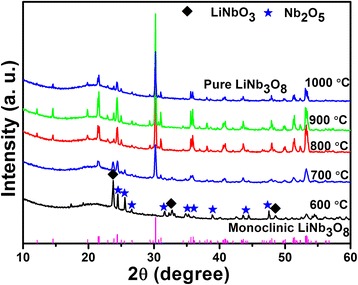
XRD patterns of the LiNb_3_O_8_ powders calcined at different temperatures for 2 h

The XRD patterns of LiNb_3_O_8_ powders calcined at different temperatures for 2 h are shown in Fig. 1. As seen in the figure, at 600 °C, the main phases are LiNbO_3_ and Nb_2_O_5_, no LiNb_3_O_8_ phase observed at all. At 700 °C, the predominant phase is LiNb_3_O_8_, with a small amount of residual LiNbO_3_, which means LiNb_3_O_8_ is more easily prepared by the hydrothermal method assisting sintering process than conventional methods [10, 11]. With the calcination temperature increase, only pure phase LiNb_3_O_8_ is observed and the phase is stable even up to 1000 °C; also, higher calcination temperature means better crystallinity and larger grain size. As shown in the figure, the phase is perfectly consistent with the JCPDF card no. 36-0307 (inserted in Fig. 1 as a reference), which is indexed to the monoclinic phase, a space group of P21/a.

**Fig. 2:**
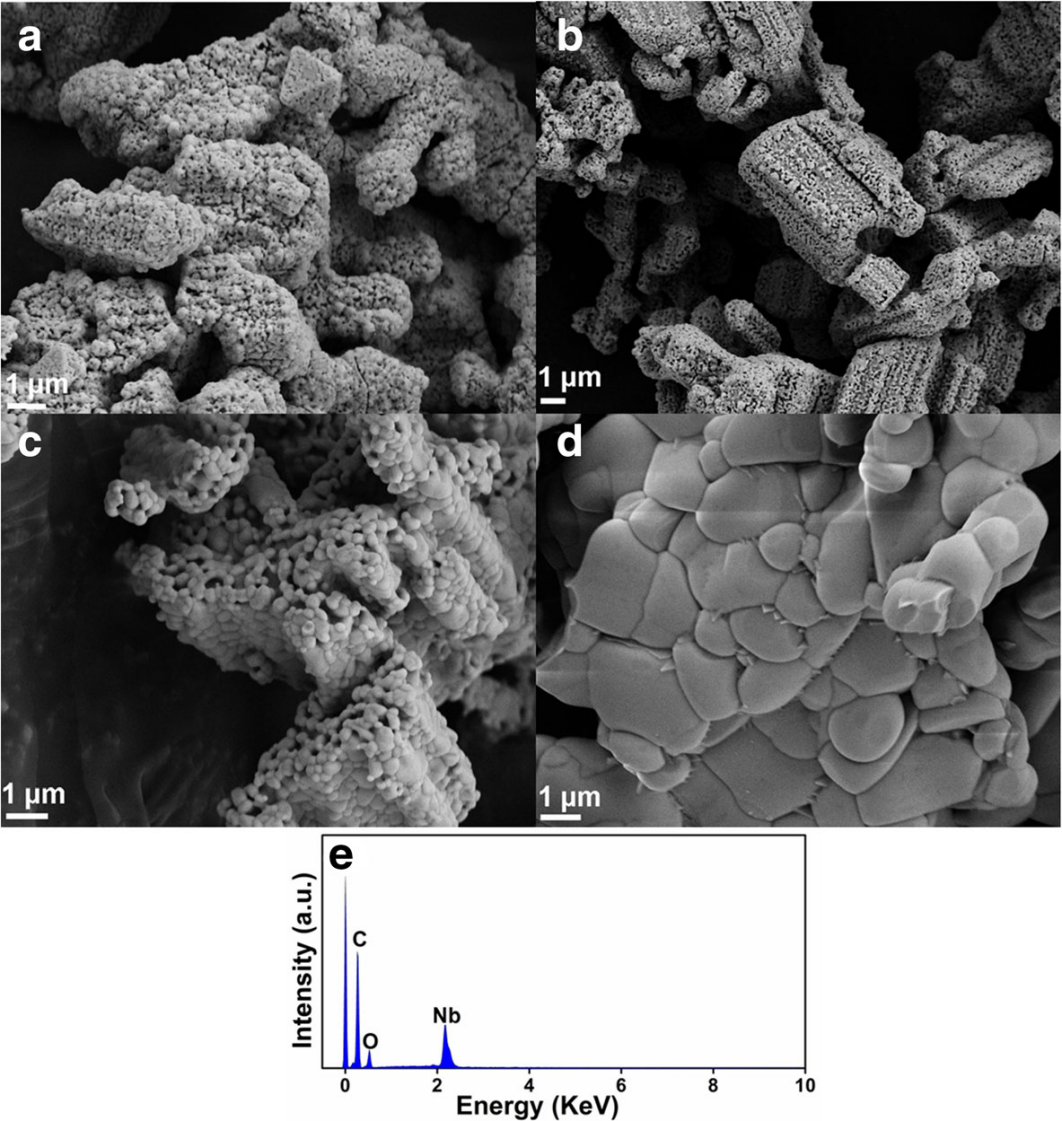
SEM images of LiNb_3_O_8_ powders calcined at **a** 700°, **b** 800°, **c** 900°, and **d** 1000 °C, respectively. **e** EDS spectrum of LiNb_3_O_8_ powders

The SEM images of LiNb_3_O_8_ powders calcined at different temperatures are displayed in Fig. 2. It can be clearly seen that at 700 and 800 °C, the particles aggregate to form hollow structures with obvious cavities. It can be attributed to the Li element volatilization during calcination process, which is beneficial to the formation of new LiNb_3_O_8_ particles and networks between the particles [15]. At the same time, the connection sites and particle shapes seem indistinct in the sample at 700 °C due to its poor crystallinity. With the calcination temperature increase, the grain size increases from ~100 nm at 700 °C to 1~3 μm at 1000 °C; the particle shapes become more obvious with improved crystallinity; the cavities become less and less with the hollow structure almost disappearing at 1000 °C. As we know, a small particle size always means high specific surface area. Both high specific surface area and good crystallinity are important factors to affect photocatalytic activity, so a trade-off must be achieved [4]. The chemical composition measured by EDS is shown in Fig. 2e. It shows the presence of C, O, and Nb elements in the synthesized LiNb_3_O_8_ powders, as Li element is undetectable.

**Fig. 3:**
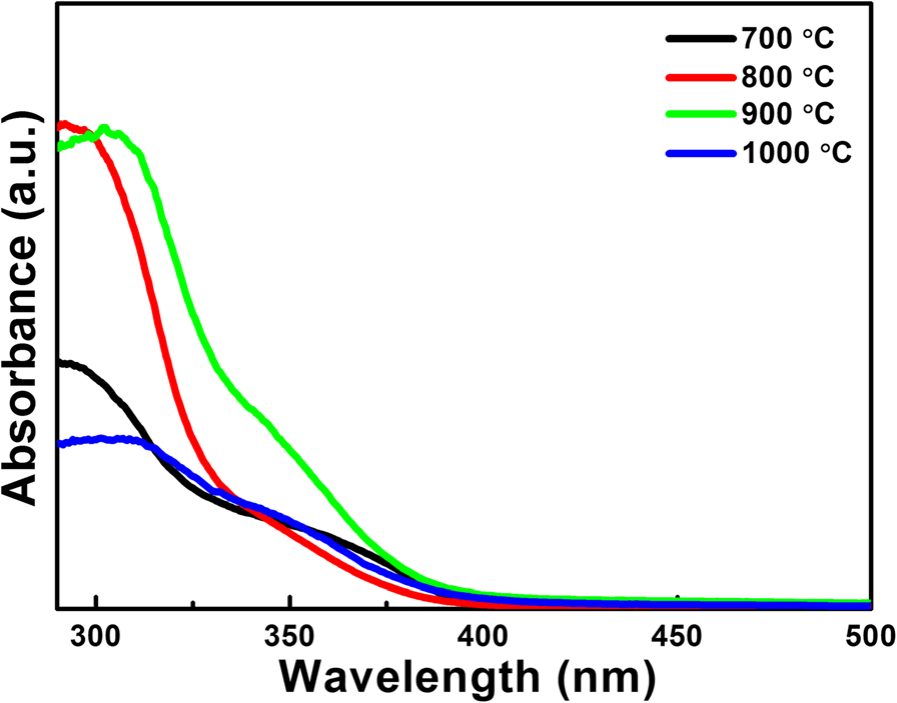
UV-vis diffuse reflectance absorbance spectra of LiNb_3_O_8_ powders

The optical properties of hollow structure LiNb_3_O_8_ powders were also measured. The UV-vis diffuse reflectance absorbance spectra of LiNb_3_O_8_ powders are recorded in Fig. 3. Using a pressed BaSO_4_ powder as the reference, the absorbance coefficient (*α*) is obtained from the diffuse reflectance spectra based on Kubelka-Munk (K-M) theory. As LiNb_3_O_8_ is the direct bandgap semiconductor [12], the bandgap (*E*_g_) can be calculated according to the relation between the absorption edge and photon energy (hv) written as follows:

1 $$ \alpha \mathrm{h}v=A{\left(\mathrm{h}v-{E}_g\right)}^{\frac{1}{2}} $$

where *A* is the absorbance constant of the semiconductors. The bandgaps of LiNb_3_O_8_ powders calcined at 700°, 800°, 900°, and 1000 °C (denoted as LNO700, LNO800, LNO900, and LNO1000, respectively) are estimated as 3.74, 3.78, 3.76, and 3.71 eV, respectively, smaller than the bandgaps reported before [12, 14]. It means the LiNb_3_O_8_ powders can only absorb UV light during photocatalytic process.

**Fig. 4:**
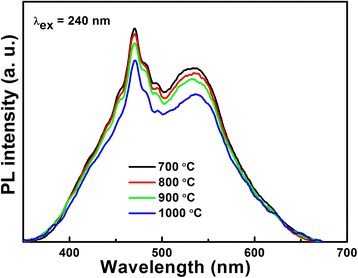
Room temperature PL spectra of the LiNb_3_O_8_ photocatalysts

The separation efficiency of photogenerated carries of the LiNb_3_O_8_ photocatalysts is investigated by PL spectra, as shown in Fig. 4. It can be clearly seen that for the LiNb_3_O_8_ photocatalysts, the intensity of PL emission peak gradually weakens. As the higher PL emission peak always corresponds to the easier combination of carriers, so the LiNb_3_O_8_ photocatalyst exhibits better surface photogenerated electron-hole separation efficiency with the increase of calcination temperature, which can be attributed to the improved crystallinity with obvious grain size growth. Especially for LNO1000, its grain size is about several micrometers, quite different from other three hollow structure LiNb_3_O_8_ powders. Though higher calcination temperature which can improve the separation efficiency of photogenerated carries increases, it also results in the large reduce of the specific surface area, which is one of the most important factors influencing the photocatalytic efficiency. The BET areas of LNO700, LNO800, LNO900, and LNO1000 are 10.7, 4.46, 0.36, and 0.23 m^2^/g, respectively; the larger surface area of LNO700 and LNO800 results from the porous and hollow structure.

**Fig. 5:**
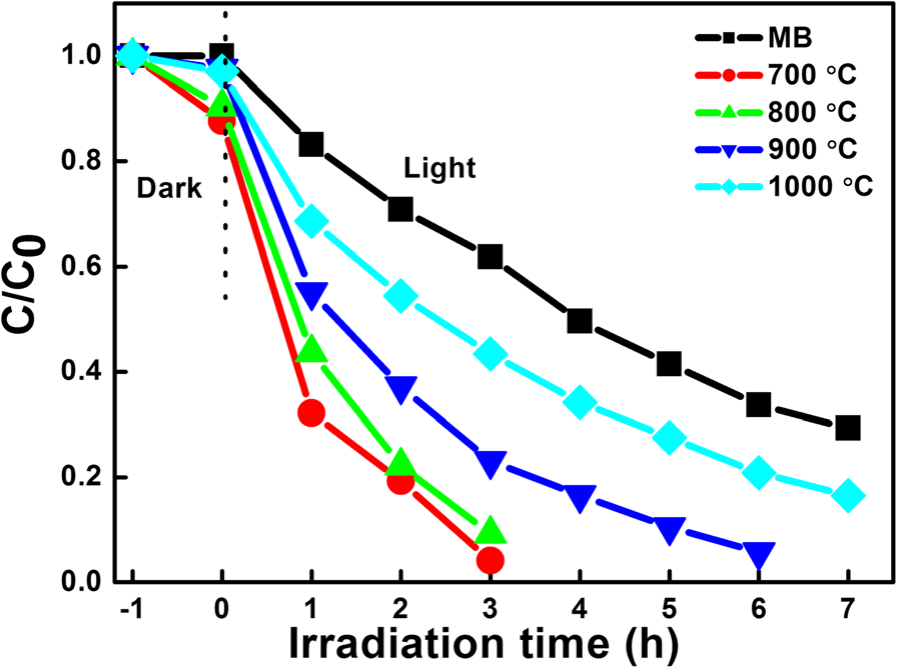
Photo-degradation of MB with respect to the irradiation time using LiNb_3_O_8_ powders exposed to UV light. Absorption ability of LiNb_3_O_8_ powders is tested after stirring for 1 h in dark to achieve the equilibrium adsorption

The photocatalytic performance of LiNb_3_O_8_ powders is evaluated by the degradation of MB under UV light irradiation, as illustrated in Fig. 5. Before the irradiation, the adsorption/desorption equilibrium is achieved in the dark to investigate the adsorption ability. It shows that LNO700 and LNO800 powders show good adsorption ability, about 14 and 10%, respectively, while only 3% for both LNO900 and LNO1000; the adsorption ability is well consistent with the morphologies of the photocatalysts shown in Fig. 2. Compared with the degradation of MB without photocatalyst, all the LiNb_3_O_8_ powders show the higher photocatalytic efficiency of degradation of MB, especially for LNO700, with only 3 h to completely decompose MB. And the TOC% of the same sample taken after a 3-h reaction time shows 83% removal of the dye organic carbons. The difference between C/C_0_ and TOC% value is mostly related to the presence of non-degradable intermediates. It means LiNb_3_O_8_ powders are efficient photocatalysts in the degradation of organic pollutants. The photocatalytic efficiency of LiNb_3_O_8_ catalysts is ranked in an order from the highest to the lowest: BNO700 > BNO800 > BNO900 > BNO1000. It can be seen that with the calcination temperature increases, the photocatalytic degradation ability decreases, which can be attributed to the morphologies change of LiNb_3_O_8_ powders: hollow structures with obvious cavities are gradually disappearing. So, hollow structures play the most important role in the degradation process, which provide a larger specific surface area and more active sites to degrade the MB molecules. For LNO700, the best photocatalytic performance may be also attributed to the synergistic effect between LiNb_3_O_8_ and LiNbO_3_. These two niobate forms can interact with each other, and photogenerated electrons can avoid recombination more efficiently [14].

**Fig. 6:**
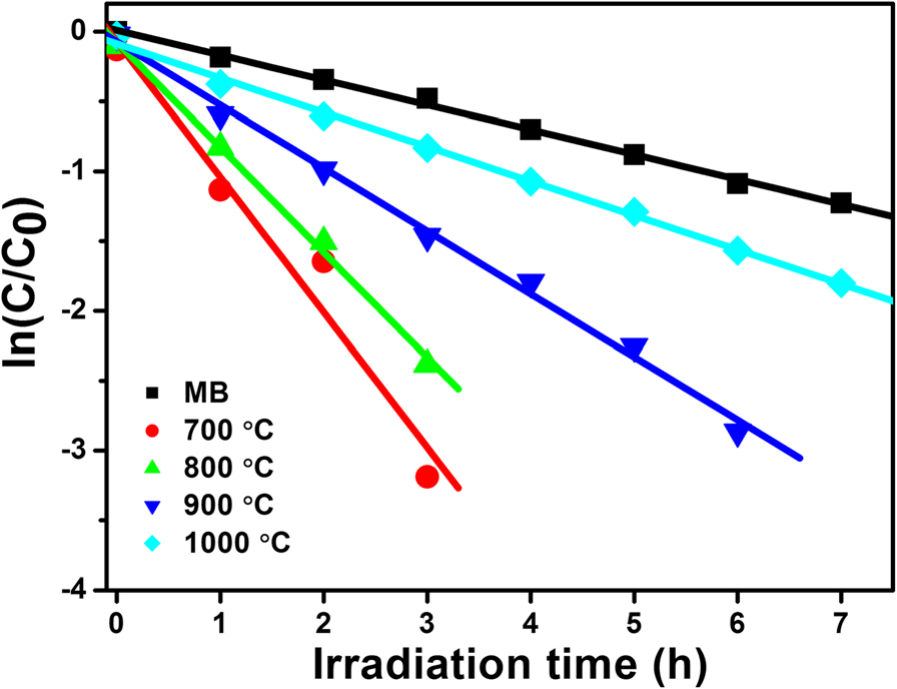
Kinetic fit for the photo-degradation of MB in the presence of LiNb_3_O_8_ powders calcined at different temperatures

The first-order rate constant (*k*) is also calculated to exhibit the photocatalytic ability of LiNb_3_O_8_ powders based on the modified Langmuir-Hinshelwood kinetics model [24], as shown in Fig. 6. The obtained *k* are 0.18, 0.97, 0.75, 0.45, and 0.25/h for MB, LNO700, LNO800, LNO900, and LNO1000, respectively. The apparent rate also shows that LNO700 with a hollow structure is the most efficient photocatalyst among them, about 4 times higher than that of LNO1000 and 5.5 times higher than that of MB without a photocatalyst.

**Fig. 7:**
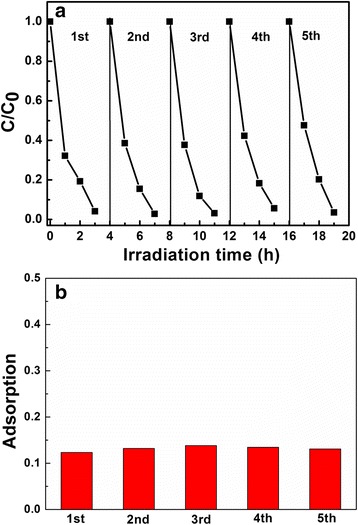
Cycling **a** photo-degradation and **b** adsorption of MB over LNO700 powder

To investigate the reusability and stability of the hollow structure LiNb_3_O_8_ photocatalyst (LNO700) for both the photocatalytic degradation and adsorption ability of MB, five cycles of photo-degradation of MB are performed, as shown in Fig. 7a, b. After five cycles of photo-degradation of MB, there shows no obvious performance loss with complete decomposition of MB in 3 h. At the same time, we firstly studied the stability of the adsorption ability of LNO700, and the results show that for each cycle, the adsorption of MB under the dark can almost keep constant. It indicates that the hollow structure of the LiNb_3_O_8_ photocatalyst is stable, which guarantees that the LiNb_3_O_8_ photocatalyst with hollow structures is an efficient photocatalyst with good reusability for practical applications.

**Fig. 8:**
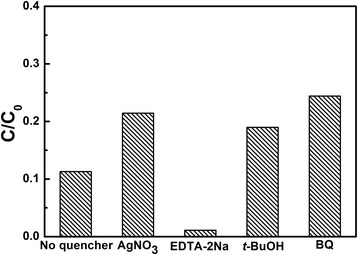
Trapping experiment of active species during the degradation of MB under UV light irradiation with the presence of LiNb_3_O_8_ catalysts

Figure 8 displays the trapping experiment of active species during the photocatalytic reaction process with LNO700 catalysts. It can be seen that the degradation of MB is obviously decreased with the addition of AgNO_3_ (a quencher of e^−^), *t*-BuOH (a quencher of ·OH) and BQ (a quencher of O_2_^·^). On the contrary, the degradation increased with the addition of EDTA-2Na (a quencher of h^+^), which means the separation of electrons and holes are promoted and more electrons are generated. Therefore, it can be concluded that e^−^, ·OH and O_2_^·^ are the main active species in the degradation process rather than h^+^. In the photocatalytic process, the photogenerated electrons (e^−^) in the conduction band transfer to the surface of the LiNb_3_O_8_ photocatalyst and reduce molecular oxygen to superoxide anion (O_2_^·^); then, the superoxide anion can react with H_2_O to form the active radicals (·OH) [25, 26]. These reactions would finally result in the degradation of MB.

**Fig. 9:**
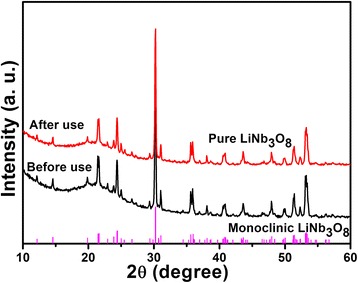
XRD patterns of LNO800 photocatalysts before and after photo-degradation of MB under UV irradiation

**Fig. 10:**
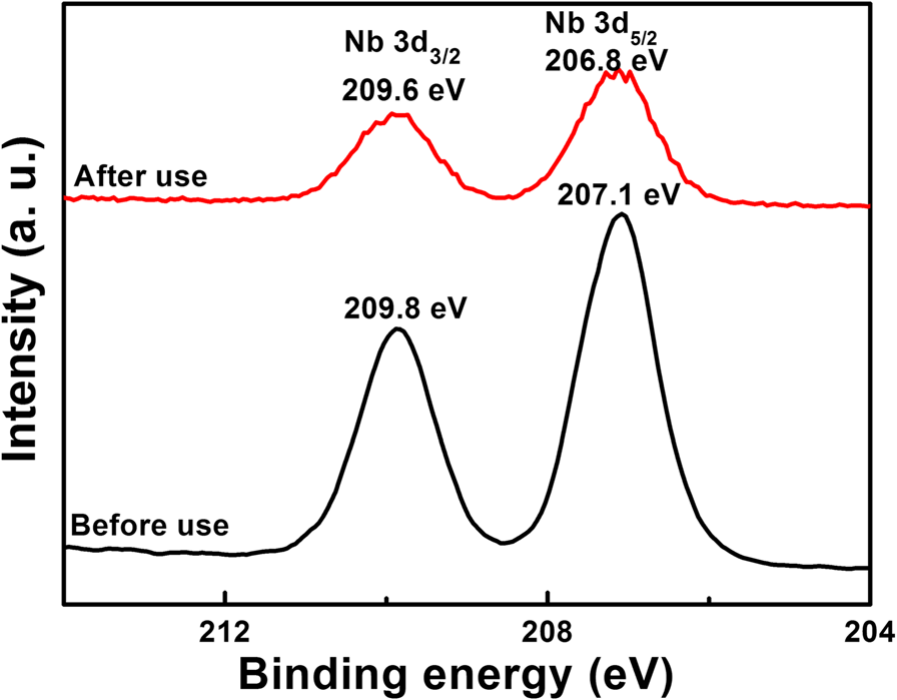
XPS spectra of Nb3d for LNO800 photocatalysts before and after photo-degradation of MB under UV irradiation

To investigate the photocorrosion of the LiNb_3_O_8_ photocatalyst, LNO800 is characterized by XRD and XPS before and after the photo-degradation of MB, as shown in Figs. 9 and 10. The XRD results show that the crystal structures of the LiNb_3_O_8_ photocatalyst varied negligibly after use, still pure LiNb_3_O_8_ without obvious impurities. However, in the XPS spectra, Nb3d peaks are shifted to lower binding energy compared to the unused LiNb_3_O_8_, indicating that partially, Nb^5+^ has been reduced and photoreduction of LiNb_3_O_8_ occurred on the surface during the photo-degradation process [15, 27–29].

## Conclusions

The hollow structure LiNb_3_O_8_ photocatalysts were prepared by the hydrothermal method assisting sintering process. The particles’ aggregation to form hollow structures with obvious cavities can be attributed to the Li element volatilization during calcination process. All the LiNb_3_O_8_ powders show high photocatalytic efficiency of the degradation of MB, especially for LNO700, with only 3 h to completely decompose MB. The photo-degradation of MB follows the pseudo-first-order kinetics, and the obtained first-order rate is 0.97/h. The larger degradation rate of LNO700 can be attributed to its hollow structure which provides a larger specific surface area and more active sites to degrade the MB molecules. The cycling test of photo-degradation and adsorption of MB over LNO700 powder indicates that the hollow structure of the LiNb_3_O_8_ photocatalyst is stable and the LiNb_3_O_8_ photocatalyst is an efficient photocatalyst with good reusability for practical applications, confirmed by the XRD and XPS tests before and after photo-degradation of MB.
